# Supplementary material for: Phylogenetic and Timescale Analysis of Barmah Forest Virus as Inferred from Genome Sequence Analysis
Source: Viruses. 2020 Jul 6;12(7):732. doi: 10.3390/v12070732 (PMC7412159; doi:10.3390/v12070732)
Supplement: Supplementary file 1 [file viruses-12-00732-s001.pdf]

viruses

MDPI

Supplementary

Phylogenetic and Timescale Analysis of Barmah Forest Virus as Inferred from Genome Sequence Analysis

Alice Michie [1], Timo Ernst [1], I-Ly Joanna Chua [2], Michael D. A. Lindsay [3], Peter J. Neville [3], Jay Nicholson [3], Andrew Jardine [3] John S. Mackenzie [2,4,5], David W. Smith [2] and Allison Imrie [1,*]

1 School of Biomedical Sciences, University of Western Australia, Nedlands 6009, Australia; alice.michie@uwa.edu.au (A.M.); timo.ernst@uwa.edu.au (T.E.)

2 PathWest Laboratory Medicine Western Australia, Perth 6000, Australia; joanna.chua@health.uwa.edu.au (I-L.J.C.); j.mackenzie@curtin.edu.au (J.S.M.); david.smith@health.wa.gov.au (D.W.S.)

3 Environmental Health Hazards, Department of Health, Perth 6000, Western Australia, Australia; michael.lindsay@health.wa.gov.au (M.D.A.L.); peter.neville@health.wa.gov.au (P.J.N.); jay.nicholson@health.wa.gov.au (J.N.); andrew.jardine@health.wa.gov.au (A.J.)

4 Faculty of Health Sciences, Curtin University, Bentley 6102, Western Australia, Australia

5 School of Chemistry and Molecular Biosciences, University of Queensland, St Lucia 4067, Australia

* Correspondence: Allison.imrie@uwa.edu.au; Tel.: +61406610730

Received: 11 May 2020; Accepted: 4 July 2020; Published: date

Abstract: Barmah Forest virus (BFV) is a medically important mosquito-borne alphavirus endemic to Australia. Symptomatic disease can be a major cause of morbidity, associated with fever, rash, and debilitating arthralgia. BFV disease is similar to that caused by Ross River virus (RRV), the other major Australian alphavirus. Currently, just four BFV whole-genome sequences are available with no genome-scale phylogeny in existence to robustly characterise genetic diversity. Thirty novel genome sequences were derived for this study, for a final 34-taxon dataset sampled over a 44 year period. Three distinct BFV genotypes were characterised (G1–3) that have circulated in Australia and Papua New Guinea (PNG). Evidence of spatio-temporal co-circulation of G2 and G3 within regions of Australia was noted, including in the South West region of Western Australia (WA) during the first reported disease outbreaks in the state's history. Compared with RRV, the BFV population appeared more stable with less frequent emergence of novel lineages. Preliminary in vitro assessment of RRV and BFV replication kinetics found that RRV replicates at a significantly faster rate and to a higher, more persistent titre compared with BFV, perhaps indicating mosquitoes may be infectious with RRV for longer than with BFV. This investigation resolved a greater diversity of BFV, and a greater understanding of the evolutionary dynamics and history was attained.

Keywords: Australia; alphavirus; arbovirus; evolutionary analysis; phylogeny

Supplementary Table 1. Metadata of viruses sequenced for this study, including the isolation location, date, species from which the virus was isolated and the NCBI accession number. Ae.; Aedes, Cx.; Culex.

Isolate Name	Location of Collection	Collection Date	Source Species	Accession Number
DC30314	Western Australia, Murray	12/12/2000	*Aedes vigilax*	MN689021
DC45960	Western Australia, Murray	11/11/2008	*Ae. camptorhynchus*	MN689022
DC56192	Western Australia, Harvey	29/1/2013	*Ae. vigilax*	MN689023
DC57911	Western Australia, Mandurah	29/10/2019	*Ae. camptorhynchus*	MN689024
EGR27629	New South Wales	2014	*Macropus giganteus*	MN689025
K60652	Western Australia, Wyndham	2006	Unknown mosquito species	MN689026
K61404	Western Australia, Derby	2006	*Ae. normanensis*	MN689027
K67171	Western Australia, Wyndham	2008	*Culex annulirostris*	MN689028
K67289	Western Australia, Parrys Creek	2008	Unknown mosquito species	MN689029
K80639	Western Australia, Willie Creek	2013	Unknown mosquito species	MN689030
KO376-1	Western Australia, Kununurra	1980	*Mansonia uniformis*	MN689031
SW26969	Western Australia, Cockburn	6/1/1993	*Cx. annulirostris*	MN689032
SW28057	Western Australia, Rockingham	4/1/1993	*Coquiellidia linealis*-like	MN689033
SW31286	Western Australia, Mandurah	5/8/1993	*Ae. camptorhynchus*	MN689034
SW35221	Western Australia, Capel	15/11/1993	*Ae. camptorhynchus*	MN689035
SW67821	Western Australia, Busselton	5/11/2001	*Ae. camptorhynchus*	MN689036
SW68009	Western Australia, Busselton	26/11/2001	*Ae. camptorhynchus*	MN689037
SW75325	Western Australia, Busselton	2/8/2005	*Ae. camptorhynchus*	MN689038
SW76326	Western Australia, Harvey	25/10/2005	*Ae. camptorhynchus*	MN689039
SW77318	Western Australia, Busselton	3/1/2006	*Ae. camptorhynchus*	MN689040
SW93518	Western Australia, Busselton	11/9/2012	*Ae. camptorhynchus*	MN689041
SW94096	Western Australia, Capel	23/10/2012	*Ae. camptorhynchus*	MN689042
SW94393	Western Australia, Capel	20/11/2012	*Ae. camptorhynchus*	MN689043
SW94457	Western Australia, Harvey	4/12/2012	*Ae. camptorhynchus*	MN689044
SW97836	Western Australia, Dardanup	4/2/2014	*Ae. alboannulatus*	MN689045
SW105045	Western Australia, Harvey	26/9/2017	*Ae. camptorhynchus*	MN689046
SW105961	Western Australia, Harvey	16/11/2017	*Ae. camptorhynchus*	MN689047
BH2193	Victoria	1974	*Cx. annulirostris*	U73745
MIDITully	Queensland	2017	*Verrallina* species	MK697273
MIDIB78	Queensland	2018	*Cx. annulirostris*	MK697274
PNG_BFV	Papua New Guinea	April 2014	*Homo sapiens*	MN115377

Supplementary Table 2. Observed amino acid substitutions that were unique to one of the three Barmah Forest virus (BFV) genotypes (G1–3). The gene region in which these substitutions were observed has been listed.

Amino Acid Substitution	Gene region	Genotype
S463P	nsP1	G1
A472E	nsP1	G1
A492V	nsP1	G2
E521D	nsP1	G1
K138Q	nsP2	G3
I268L	nsP2`	G3
T484M	nsP2	G1
R537K	nsP2	G3
L764F	nsP2	G1
S798N	nsP2	G3
G244S	nsP3	G1
L247V	nsP3	G3, except SW94457 - had deletion at this site
V373I	nsP3	G3
T384I	nsP3	G3
V737I	nsP3	G3
S443A	nsP3	G3
H92R	nsP4	G1
P93S	nsP4	G1
R111L	nsP4	G2
K118E	nsP4	G1
R167K	nsP4	G1
K422R	nsP4	G3
S90P	C	G1
D52E	E3	G2
A221T	E2	G1
V281A	E2	G1
N303S	E2	G2
F382L	E2	G3
N72S	E1	G1
V260I	E1	G2

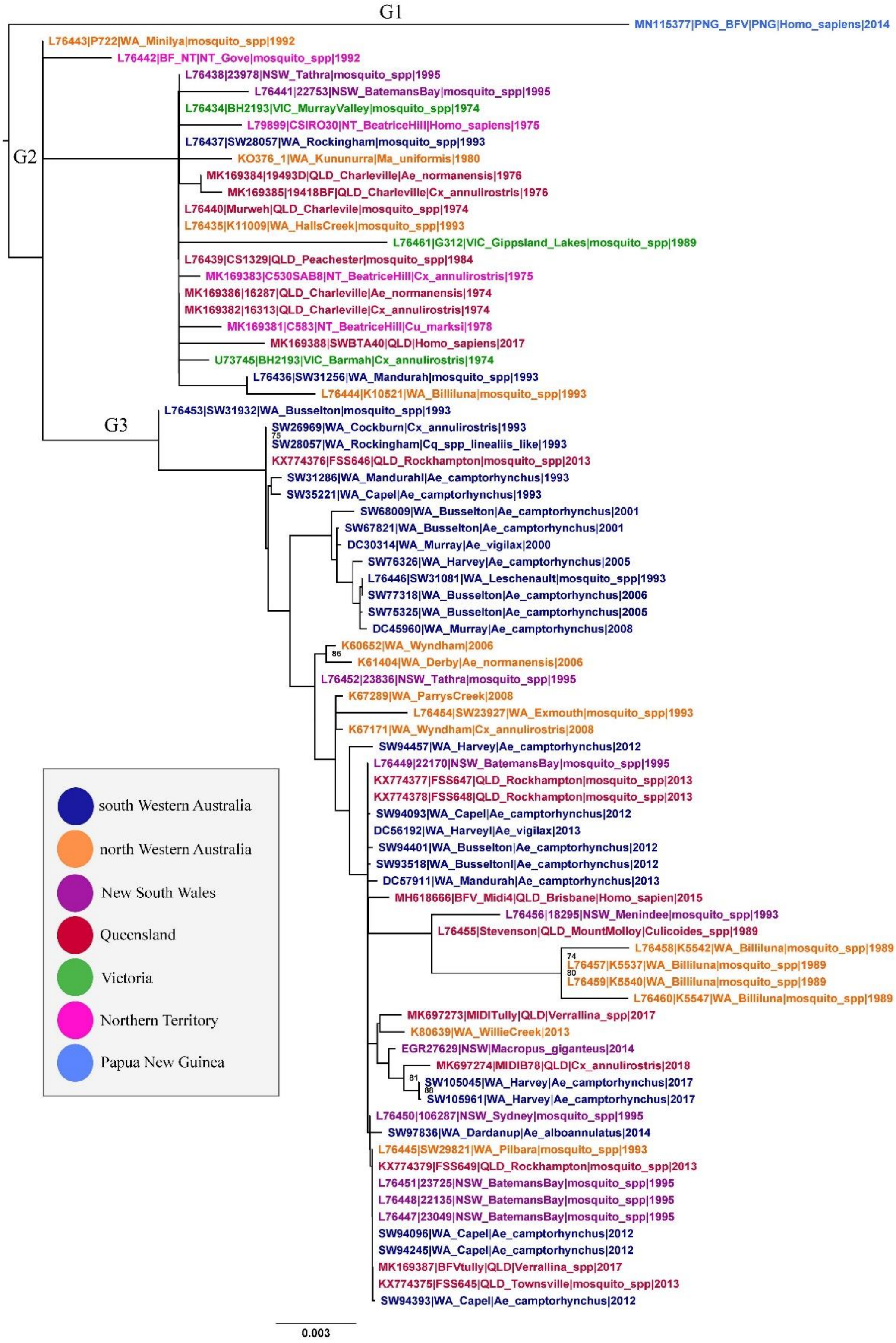

Supplementary Figure 1. Midpoint rooted maximum likelihood phylogeny of the 34-taxon Barmah Forest virus dataset, aligned with all available whole or partial E2 gene sequences that were geographically defined. Taxa are coloured for their geographical origin (see key). Bootstrap values >70% are shown above supported nodes (*).

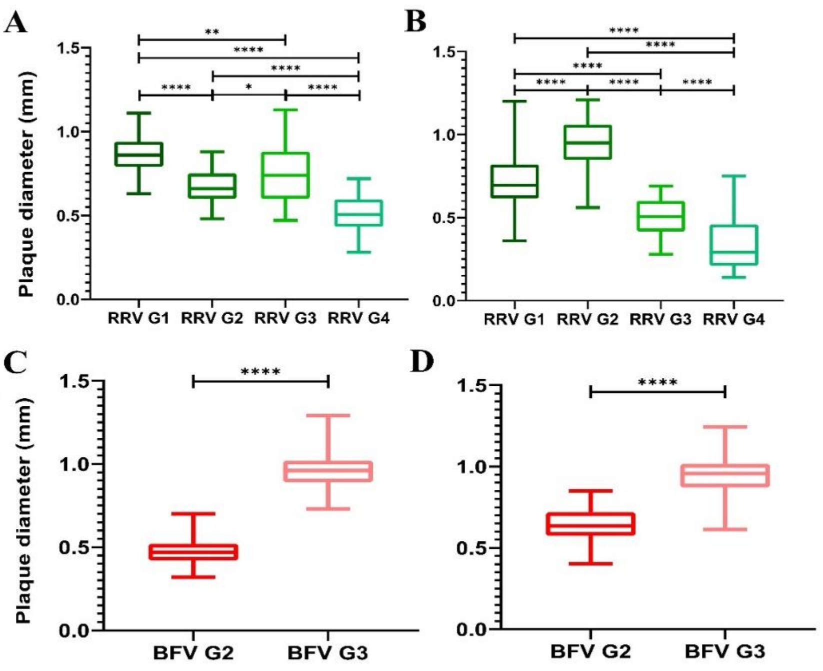

Supplementary Figure 2. Size comparison of plaques produced from C6/36 and Vero cell culture supernatant, collected during infection with representatives of Ross River virus (G1–4) and Barmah Forest virus (G2–3) genotypes. All culture supernatant was inoculated onto Vero cell monolayers for plaque assay quantification. **A**) Comparison of plaque size between RRV G1–4 from C6/36 cell supernatant **B**) Comparison of plaque size between RRV G1–4 from Vero cell supernatant **C**) Comparison of plaque size between BFV G2–3 from C6/36 cell supernatant **D**) Comparison of plaque size between BFV G2–3 from Vero cell supernatant. Significance values are shown above comparative datasets ($p \leq 0.0001$; ****, $p \leq 0.001$; ***, $p \leq 0.01$; **, $p \leq 0.05$; *).

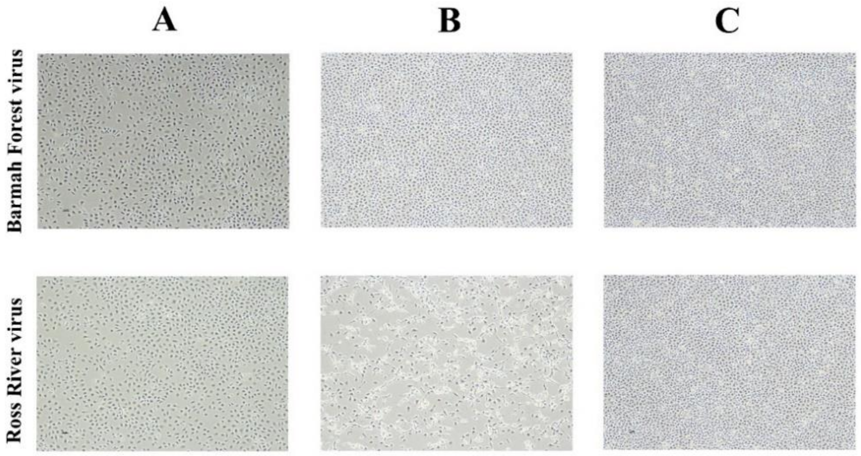

Supplementary Figure 3. C6/36 cells at different time points of Ross River virus (lower panel) and Barmah Forest virus (upper panel) infection. **A)** presents cells prior to virus inoculation. **B)** presents cells 48-hours post infection. **C)** presents the virus-free negative control cells at 48-hours post-mock infection. Data shown is infection with G1 of RRV and G3 of BFV, as representatives. These observations were consistent between all RRV and BFV variants studied
